# Collost Bioplastic Collagen Material for the Treatment of Burns

**DOI:** 10.17691/stm2020.12.1.12

**Published:** 2020

**Authors:** L.I. Budkevich, G.V. Mirzoyan, R.B. Gabitov, M.A. Brazol, P.V. Salistyj, Y.V. Chikinev, A.A. Shmyrin, A.V. Glutkin

**Affiliations:** Professor, Chief Researcher, Department of Combustiology and Reconstructive Plastic Surgery, Research Institute of Pediatric Surgery, Pirogov Russian National Research Medical University, 1 Ostrovitianova St., Moscow, 117997, Russia; Head of the Burn Center, Children’s City Clinical Hospital No.9 named after G.N. Speransky, 29 Shmitovskiy Proezd, Moscow, 123317, Russia; Head of the 2^nd^ Burn Unit, Children’s City Clinical Hospital No.9 named after G.N. Speransky, 29 Shmitovskiy Proezd, Moscow, 123317, Russia; Pediatric Surgeon, the 2^nd^ Burn Unit, Children’s City Clinical Hospital No.9 named after G.N. Speransky, 29 Shmitovskiy Proezd, Moscow, 123317, Russia; Scientific Consultant, Department of Surgical Diseases and Clinical Angiology, A.I. Yevdokimov Moscow State University of Medicine and Dentistry, 20/1 Delegatskaya St., Moscow, 127473, Russia; Head of the Burn Unit; Deputy Chief Surgeon, Children’s City Hospital No.1, 14 Avangardnaya St., Saint Petersburg, 198205, Russia; Head of the Burn Unit, Children’s City Clinical Hospital No.9, 51 Reshetskaya St., Ekaterinburg, 620134, Russia; Professor, Head of the Department of Hospital and Pediatric Surgery, Novosibirsk State Medical University, 52 Krasnyy Prospekt, Novosibirsk, 630091, Russia; Head of the Unit of Combustiology, Novosibirsk State Regional Clinical Hospital, 130 Nemirovicha-Danchenko St., Novosibirsk, 630087, Russia; Associate Professor, Department of Pediatric Surgery, Grodno State Medical University, 80 Gor’kogo St., Grodno, 230009, Republic of Belarus

**Keywords:** mosaic burns, thermal burns, type 1 collagen, Collost

## Abstract

**The aim of the study** was to evaluate the efficacy and safety of the Collost bioplastic material in the treatment of borderline and mosaic burns.

**Materials and Methods:**

We conducted a prospective multicenter study, which included 94 patients aged 1 to 12 years with thermal skin burns (grade II–III by ICD-10). Patients were divided into four groups. In groups 1–3, various forms of the Collost bioplastic material were used (group 1 — 7% gel, group 2 — membranes, group 3 — powder) in combination with hydrocolloid dressings containing Ag^+^ ions. Patients of the control group (group 4) underwent the traditional local conservative treatment using hydrocolloid dressings alone. Concomitant therapy was similar in all of the participating centers. The total follow-up period was 4 weeks from the date of burn injury.

**Results:**

On day 14, there were 23 cases (92%) of complete epithelization in group 1, 13 cases (68.4%) — in group 2, 21 cases (78%) — in group 3, and 9 cases (39.1%) — in group 4. The data from groups 1 and 3 significantly differed from those in control (p<0.05). The epithelialization of the burned skin in the Collost groups (7% gel and powder) was on average one week faster compared to the control.

**Conclusion:**

The Collost bioplastic material (in the form of gel or powder) in combination with hydrocolloid dressings can be a functional and inexpensive alternative to autografts in the treatment of borderline and mosaic burns.

## Introduction

Every year, 450 thousand people with fresh burns turn for medical assistance in the Russian Federation. About 70% of them, have minor superficial burns and undergo treatment in the outpatient clinic. The rest of the patients have deep and borderline burns; most often, such injuries are presented by children under the age of 5 years [1].

According to the international classification of diseases ICD-10, burns are classified as superficial (grade I epidermal burns), borderline (grade II dermal burns), and deep (grade III burns). With grade II burns (borderline), some of the skin components remain intact; those provide for epithelization of the burned area within 18–21 days. Notably, only small-sized deep burns can heal via this mechanism of marginal epithelization. More often, grade II borderline burns involve skin depigmentation and post-burn scars, especially after “mosaic” burns (grade II burns with isolated areas of grade III skin lesions). The currently recommended treatment for grade II borderline burns is based on conservative therapy in humidified conditions using special wound dressings. In some cases, it is proposed to use biological wound dressings alone or in combination with auto-dermoplasty [1].

Collagen-based biological coatings are able to effectively control the wound exudate, inactivate proteinases, protect endogenous and exogenous growth factors from degradation, and serve as a bioplastic matrix for the formation of patient’s own connective tissue [2–4]. Such is the Collost coating based on native unreconstructed bovine type 1 collagen. The clinical efficacy of this coating when applied as 7% gel or membranes was earlier evaluated in 6 children aged 11 to 20 months with thermal skin burns of 3 to 12% of the body surface [5]. A faster tissue epithelization in comparison with the traditional conservative treatment was found. No allergic reactions were noted [5]. In addition, Collost membranes of 60×50×1.5 mm accelerated the healing of residual wounds resulted from deep burns in adult patients [6]. Another study showed that Collost membranes stimulated the granulations of long-term non-healing residual wounds and thus prepared the affected skin for subsequent auto-dermoplasty [7].

**The aim of this study** was to evaluate the efficacy and safety of the Collost bioplastic material in the conservative treatment of borderline and mosaic burns.

## Materials and Methods

A prospective multicenter study in 94 patients aged 1 to 12 years with thermal burns (99% of them with hot liquid) was conducted. During the first visit, the condition of 53 children (56%) was defined as moderate to severe, the rest of the children were in a satisfactory condition.

All patients presented with mosaic burns, mainly grade II–IIIA with small sections grade IIIB burns, which corresponds to the grade II–III burns according to ICD-10. The average burn duration was 5.2±1.7 days before the study began.

The study was conducted in accordance with the Helsinki Declaration (2013) and approved by the local ethics committees of the participating medical centers. Informed consent was obtained from patients’ parents in accordance with the Federal Law “Fundamentals of the Legislation of the Russian Federation on the Protection of Health of Citizens” (2011).

The burns were located as follows: the neck — 2 children, the front surface of the trunk and/or shoulder — 24 patients, the front surface of the trunk and thigh — 1, the back surface of the trunk/back surface of the trunk and shoulder — 7, the shoulder and/or forearms — 20, forearms and/or hands — 11, hips and/or lower legs — 11, lower legs and feet — 4, and the feet — 14.

The patients were divided into four groups, comparable by sex, age, body mass index, characteristics of the burns and skin lesions (Table 1).

**Table 1:** Characteristics of the study groups

Indicators	Group 1 (n=25)	Group 2 (n=19)	Group 3 (n=27)	Group 4 (n=23)	Statistical significance
Sex (abs. number/%):					
male	14/56	16/84	14/52	16/70	p=0.108
female	11/44	3/16	13/48	7/30	
Age (years):					χ^2^=1.7
M±SD	2.2±2.6	2.4±2.8	3.2±3.7	3.1±3.6	df=3
Me [25; 75]	1.1 [1; 2]	1.1 [1; 2]	1.3 [1; 3]	1.3 [1.0; 4.3]	p=0.642
Body mass index (kg/m^2^):					χ^2^=0.122
M±SD	17.4±4.1	17.2±2.7	18.1±4.7	17.3±2.4	df=3
Me [25; 75]	16.4 [15.7; 18.8]	16.8 [15.7; 18.0]	16.8 [15.4; 19.7]	17.5 [15.0; 18.8]	p=0.989
Burn duration (days):					χ^2^=7.4
M±SD	5±1	6±3	5±1	5±1	df=3
Me [25; 75]	5 [5; 5]	6 [4; 9]	5 [5; 6]	4 [3; 5]	p=0.1
The burned skin area (cm^2^):					χ^2^=5.5
M±SD	42.5±41.6	37.1±40.8	44.0±26.6	47.7±39.2	df=3
Me [25; 75]	27.1 [9.1; 47.6]	18.9 [8.8; 44.0]	40.8 [16.0; 45.7]	47.0 [11.5; 52.3]	p=0.139

Note: independent nonparametric samples were compared using the Kruskal–Wallis test.

In groups 1–3, various forms of the Collost biological coating and hydrocolloid dressings with Ag^+^ ions were used. In group 4 (control), surgical treatment of burn wounds was performed and hydrocolloid dressings with Ag^+^ ions were applied.

The total follow-up period was 4 weeks from the date of burn injury. The patients underwent examinations on days 5, 9, 14, and 28.

In addition to the standard clinical methods, the wound surface area was measured at each patient visit using the Appendix V2F software. The wound healing was assessed using the Photographic Wound Assessment Tool [8] in our modification (mPWAT). Thus, the mPWAT scale included four parameters (condition of the wound edges, skin color around the wound, type of the granulation tissue, and degree of epithelization) determined from the photograph. Scores from 0 to 4 were assigned to each parameter. The final mPWAT score for a given wound was calculated by adding up these 4 scores. Thus, the range of possible results could vary from 0 to 16, while 0 points corresponding to a fully healed wound.

***Methodology of using the Collost bioplastic material.*** Patients of group 1 (n=25) were treated by applying 7% Collost gel uniformly upon the wound surface, which was then covered with an Ag^+^ hydrocolloid dressing. The dressing was changed every 3–4 days.

In group 2 (n=19), the wound defect was covered with a Collost membrane sized at 60×50×1.5 mm. The membrane was preliminarily kept in a warm (38°C) saline solution for 15 min, then perforated to drain the exudate and adjusted to the wound size, and then placed on the wound. A hydrocolloid dressing with Ag^+^ ions was applied on top of the Collost membrane. The membrane was tightly attached to the wound surface and kept wet throughout the entire period of treatment. To tightly fit the membrane between the wound surface and the hydrocolloid dressing, a tissue-based adhesive bandage could be used. The dressing was changed every 3–4 days.

In group 3 (n=27), an even layer of Collost powder was spread over the wound surface. A hydrocolloid dressing with Ag^+^ ions was applied on the top. The dressing was changed every 3–4 days.

***Statistical analysis*** was performed using Statistica 6.0 and SPSS for Windows (SPSS Inc., USA). The numeric results are presented as M±SD or Me [25; 75] in accordance with the type of data distribution. For pairwise comparison of interdependent samples, the Wilcoxon two-sample criterion was used; for pairwise comparison of independent samples, the Mann–Whitney criterion was used. Multiple comparisons of independent nonparametric samples were performed using the Kruskal–Wallis test, and multiple comparisons of interdependent nonparametric samples were performed using the Friedman criterion. The relationship between the qualitative values was studied using the contingency tables with the χ^2^ criterion or the two-sided Fisher exact test (if the expected values in the contingency table were <5). The value of p<0.05 was taken as the level of statistical significance.

## Results

**Figure 1:**
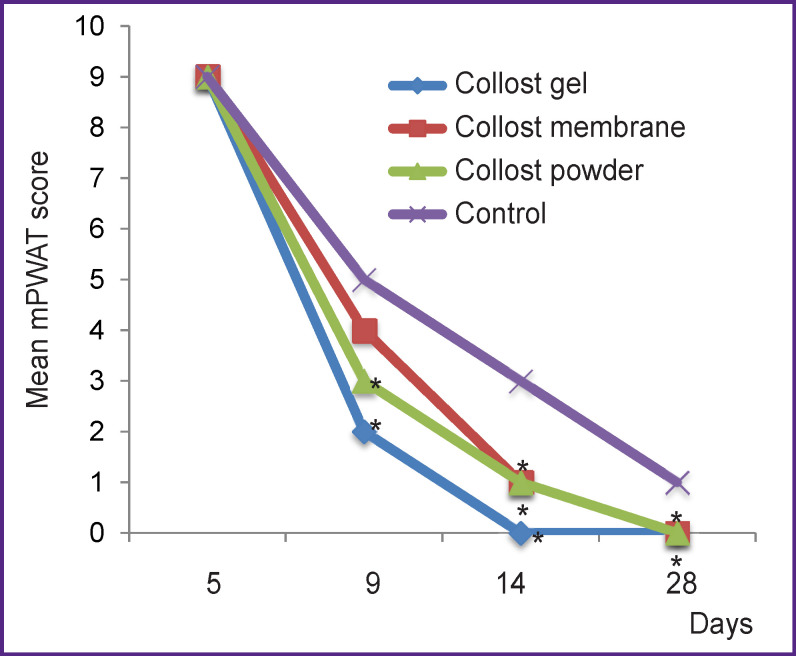
Change in the total mPWAT scores in the study groups over time * p<0.05 compared with the control group

Table 2 and Figure 1 illustrate the process of burn epithelization (according to the mPWAT scale) as recorded from the 1^st^ to the 4^th^ visit. On the 3^rd^ visit (day 14), an improvement in the healing process of burn wounds was noted in groups 1–3 but not in the control group (p<0.05). Upon further observation (4^th^ visit, day 28), improved healing of burns was detected in group 1 (7% Collost gel) and group 3 (Collost powder) as compared with control (p<0.05).

**Table 2:** Change in the mean mPWAT scores in the study groups (M±SD)

Visits	Group 1 (n=25)	Group 2 (n=19)	Group 3 (n=27)	Group 4 (n=23)
1	9±2	9±1	9±1	9±1
2	2±2	4±4	3±3	5±3
3	0±1	1±2	1±2	3±4
4	0±0	0±0	0±0	1±3

Note: zero is complete wound healing.

On day 14, complete epithelization was recorded in 23 patients (92%) of group 1, in 13 patients (68.4%) of group 2, in 21 patients (78%) of group 3, and only 9 patients (39.1%) of group 4. Statistically significant differences (p<0.05) from control were found in patients of group 1 (Collost gel) and group 3 (Collost powder).

In groups 1, 2, and 3, the median number of days past from the start of the treatment to complete wound epithelialization was 7 [3; 9], 8 [4; 11], and 9 [4; 13], respectively; in the control group, this number was greater — 15 [4; 22] days. Statistically significant differences were observed between group 1 (Collost gel) and control (p<0.05).

Concomitant therapy was similar in all the participating centers. On the 1^st^ day of admission, patients underwent mechanical treatment of burn wounds, removal of necrotic epidermis and loose fibrin threads. In addition to the investigational Collost biomaterial and hydrocolloid dressings with silver ions, routine materials for local wound treatment were used. Among them, there were furacilin, Prontosan-gel, and 0.02–0.05% chlorhexidine solution. In some cases, systemic infusion and antibacterial therapy (sulperason, ceftriaxone, cefuroxime, augmentin) were initiated; non-steroidal anti-inflammatory and antihistamine medications were also used.

**Figure 2:**
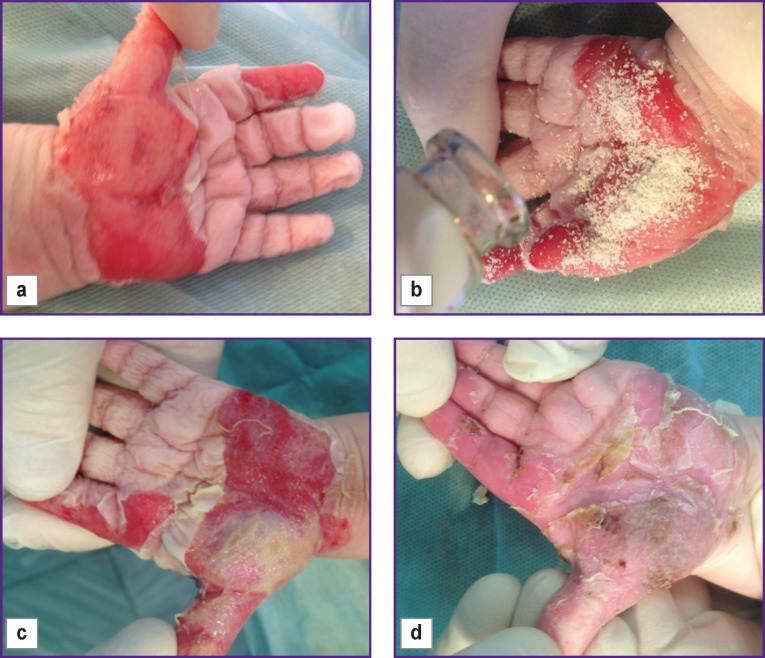
Girl (1 year old), hand burned in boiling water, grade II–III burn (ICD-10): (a) 1 day burn; (b) day 5, applying Collost powder; (c) day 9, wound granulation; (d) day 14, complete epithelization

The complete epithelization of grade II–IIIA mosaic burns with isolated areas of grade IIIB burns occurred (on average) one week faster under the Collost treatment either as powder (Figure 2) or 7% gel as compared to the traditional conservative wound treatment.

On average, the process of epithelization developed by 4.25 cm^2^/day in the Collost treated patients, as compared with 3.0 cm^2^/day in control.

In summary, using the Collost medical material in the form of powder or 7% gel made it possible to shorten the time needed for complete wound epithelization and healing by about 1.4-fold as compared with the traditional therapy. Due to the longer epithelization period, patients in the control group had more frequent changes of hydrocolloid dressings. During the study, no adverse events were observed.

## Conclusion

Of the currently available technologies for treating burn wounds, the Collost, material based on native bovine type 1 collagen, is one of the most effective, safe and economically viable. Collost formulated as powder or 7% gel (in combination with hydrocolloid dressings) reduces the time required for complete epithelization of mosaic burns; the method can become a functional and inexpensive alternative to auto transplants. Further development of novel technologies for regenerative medicine is needed to improve the clinical outcome of burn injury by reducing the time of epithelization and preventing the formation of rough scars and severe contractures.
